# Chemical Composition and Antioxidant Activity of Thyme, Hemp and Coriander Extracts: A Comparison Study of Maceration, Soxhlet, UAE and RSLDE Techniques

**DOI:** 10.3390/foods9091221

**Published:** 2020-09-02

**Authors:** Sara Palmieri, Marika Pellegrini, Antonella Ricci, Dario Compagnone, Claudio Lo Sterzo

**Affiliations:** Faculty of Bioscience and Technology for Food, Agriculture and Environment, University of Teramo, Via R. Balzarini 1, 64100 Teramo, Italy; spalmieri@unite.it (S.P.); mpellegrini@unite.it (M.P.); dcompagnone@unite.it (D.C.); closterzo@unite.it (C.L.S.)

**Keywords:** ultrasound assisted extraction—UAE, rapid solid-liquid dynamic extraction—RSLDE, gas chromatography-mass spectrometry—GC-MS, antioxidants, *C. sativa*, *T. vulgaris*, *C. sativum*

## Abstract

Appropriate and standardized techniques for the extraction of secondary metabolites with interesting biological activity from plants are required. In this work, a comparison of different conventional and unconventional extraction techniques (maceration—M, Soxhlet—S, ultrasound assisted extraction—UAE, and rapid solid-liquid dynamic extraction—RSLDE) was investigated. Bioactive compounds were extracted from *Thymus vulgaris* L. (thyme), *Cannabis sativa* L. (industrial hemp) and *Coriandrum sativum* L. (coriander) and chemically characterized for their volatile fraction and polyphenolic content by means of gas chromatography-mass spectrometry (GC-MS) and high performance liquid chromatography-ultraviolet (HPLC-UV). Linalool (48.19%, RSLDE) and carvacrol (21.30%, M) for thyme, caryophyllene (54.78%, S) and humulene (14.13%, S) for hemp, and linalool (84.16%, RSLDE) for coriander seeds were the main compounds among terpenes, while thyme was the richest source of polyphenols with rosmarinic acid (51.7 mg/g dry extract-S), apigenin (7.6 mg/g dry extract-S), and luteolin (4.1 mg/g dry extract-UAE) being the most abundant. In order to shed light on their potential as natural food preservatives, the biological activity of the extracts was assessed in terms of antioxidant activity (2,2′-azino-bis(3-ethylbenzothiazoline-6-sulphonic acid—ABTS˙^+^, ferric reducing antioxidant power—FRAP, 2,2-diphenyl-1-picrylhydrazyl—DPPH˙ assays) and phenolic content (Folin–Ciocâlteu method). For thyme, Soxhlet extracts showed best performances in FRAP and ABTS˙^+^ assays (74 mg TE/g dry extract and 134 mg TE/g dry extract, respectively), while Soxhlet and RSLDE extracts recorded similar activity in DPPH˙ (107–109 mg TE/g dry extract). For hemp and coriander, indeed, RSLDE extracts accounted for higher antioxidant activity as evidenced by FRAP (80 mg TE/g dry extract and 18 mg TE/g dry extract, respectively) and ABTS˙^+^ (557 mg TE/g dry extract and 48 mg TE/g dry extract, respectively) assays. With respect to DPPH˙, the best results were observed for UAE extracts (45 mg TE/g dry extract and 220 mg TE/g dry extract, respectively). Our findings suggest that all the investigated techniques are valid extraction methods to retain bioactive compounds and preserve their activity for application in food and pharmaceutical formulations. Among them, the innovative RSLDE stands out for the slightly higher antioxidant performances of the extracts, coupled with the facility of use and standardization of the extraction process.

## 1. Introduction

Plant bioactive compounds are defined as secondary plant metabolites capable of exerting a positive effect on animal or human health. Secondary metabolites are produced within the plants beyond the primary biosynthetic and metabolic routes of compounds [1]. These components are not needed for plant basic metabolism, can be regarded as products of biochemical “sidetracks” in the plant cells, and can cover important functions in living plants. Polyphenols, for example, can protect plants against free radicals generated during photosynthesis. Terpenoids may attract pollinators or seed dispersers or inhibit competing plants, whereas alkaloids usually ward off herbivore animals or insect attacks.

Among the best-known bioactive compounds, polyphenols and terpenes can delay or inhibit the oxidation of lipids or other biomolecules, and, thus, prevent or repair the damage of human cells caused by oxygen [2,3]. The importance of these components has been emphasized in the last years. The ever-increasing consumer sensibility to the consumption of food with lower content of synthetic chemical products and the loss of efficacy of common preservatives, due to the development and diffusion of resistant bacteria, have led to increasing research activities regarding the extraction and the evaluation of the efficacy of natural antioxidants [4,5].

The use of plant bioactive compounds as antioxidants in different commercial sectors, such as the pharmaceutical, food, and chemical industries, needs an appropriate and standardized extraction technique [6]. Extraction is the first step of any plant chemical component study and plays a significant and crucial role. The efficiency of conventional and non-conventional extraction methods strongly depends on the input parameters, the nature of the plant matrix, the chemistry of bioactive compounds, and the operator expertise [7,8].

Traditional methods, like maceration, percolation, and Soxhlet, are known to have some limits such as time and solvent consumption, and decomposition of heat sensitivity bioactive compounds [8]. However, Soxhlet technique is still common in laboratories and industries being involved in a wide variety of official methods [9]. Recently, the need of enhancing the biological activity of plant extracts has led to the development of unconventional extraction methods. Among the latter, microwave assisted extraction (MAE), supercritical fluid extraction (SFE), ultrasound assisted extraction (UAE), and rapid solid–liquid dynamic extraction (RSLDE) are the most interesting [10,11,12].

In UAE, the propagation of ultrasonic waves through a liquid medium damages plant wall, resulting in an improvement in solvent penetration; thus, bioactive components can be extracted in minutes. Therefore, with respect to conventional methods, UAE has the advantage of reducing the extraction process time and energy consumption retaining high efficiency [13,14].

The RSLDE, performed by Naviglio Extractor^®^, can be considered among the “greenest” strategies, operating at room temperature, with a minimum waste of energy and solvents. Naviglio’s principle is based on generating, with a suitable solvent, a negative pressure gradient between the internal and external sides of a solid matrix containing extractable material, followed by a sudden restoration of the initial equilibrium conditions. This process induces the forced extraction of the compounds not chemically linked to the main structure of the solid [15].

Scientific literature presents several works about RSLDE comparison with other extraction techniques. However, few records of this comparison are aimed at food preservation [16,17,18,19]. The present work focuses on the comparison of different conventional and unconventional extraction techniques (maceration, Soxhlet, UAE, and RSLDE), to obtain extracts suitable for food preservation. Three aromatic species were investigated: *Thymus vulgaris* L., *Cannabis sativa* L., and *Coriandrum sativum* L. The obtained extracts were chemically characterized, and their biological activity was assessed in terms of antioxidant activity.

## 2. Materials and Methods

### 2.1. Plant Material

Plants were open field cultivated in Abruzzo’s territory starting from certified seeds. Dry inflorescences of *Cannabis sativa* ‘Futura 75’ (hemp), dry apical stems and leaves of *Thymus vulgaris* (thyme) and seeds of *Coriandrum sativum* (coriander) were obtained from a local farmer (Hemp Farm Italia, Tortoreto (TE), Azienda Agricola Luigi Barlafante, Roseto degli Abruzzi (TE), and Mediterranea Sementi, Sant’Atto (TE), respectively).

Inflorescences of hemp were collected during the flowering period (September), let dry in a dark room at room temperature (20–25 °C), with controlled relative humidity (45–55%), and stored in the same conditions until processing. Little branches of *T. vulgaris* were collected during the balsamic period (June), dried on the field, and stored in a dry and darkroom until processing. Seed heads of *C. sativum* were cut off when the plant began to turn brown, put in a paper bag, and hanged. After drying, seeds were collected and stored in sealed bags.

### 2.2. Chemicals

Ethanol absolute was obtained from Carlo Erba (Milan, Italy). Acetic acid, acetonitrile, methanol, and water (high performance liquid chromatography—HPLC grade) were purchased from VWR (Milan, Italy).

α-pinene, β-pinene, linalool, β-myrcene, terpinolene, caryophyllene, humulene, and β-bisabolene, gallic acid, *p*-OH benzoic acid, chlorogenic acid, vanillic acid, caffeic acid, syringic acid, ferulic acid, and rosmarinic acid (from Sigma-Aldrich, Darmstadt, Germany) standards were employed. Working standard mixtures were prepared by appropriate dilution of the standards in methanol. All solutions were stored at −20 °C in the dark.

Folin–Ciocâlteu’s reagent, 6-hydroxy-2,5,7,8-tetramethylchroman-2-carboxylic acid (Trolox), 2,2-diphenyl-1-picrylhydrazyl (DPPH˙), and 2,2′-azino-bis(3-ethylbenzothiazoline-6-sulphonic acid) diammonium salt (ABTS˙^+^) were purchased from Sigma-Aldrich (Darmstadt, Germany). Sodium carbonate, potassium persulfate, potassium hexacyanoferrate(III), trichloroacetic acid, ferric chloride, and potassium phosphate monobasic were obtained from Carlo Erba (Milan, Italy).

### 2.3. Extractions

Before extraction, the samples were homogenized by trituration with a chopper (Kenwood Quad Blade CH580 Chopper, Kenwood Limited, Havant, UK) 3 times and then crushed with a mortar. Trituration time was as follows: hemp inflorescences, 10 s; thyme leaves and little stems, 20 s; coriander seeds, 15 s.

Extracts were obtained both with conventional methods, as maceration and Soxhlet, and using the unconventional UAE and RSLDE. RSLDE and Soxhlet extracts were produced with two commonly utilized total time extraction processes: 2 and 6 h.

The extracts were all collected in flasks, filtered, and brought to dry by Rotavapor Steroglass S.r.l. (Perugia, Italy).

The extraction yields were calculated according to the equation:

$$Yield\;(\%\;w/w)\;=\;\frac{mass\;dried\;extract(g)}{mass\;dried\;matrix(g)}\;\times \;100$$

The results were expressed as the average of two replicates of the extraction.

#### 2.3.1. RSLDE Extraction

RSLDE technique was performed using Naviglio Extractor^®^ (Atlas Filtri, Padua, Italy), using the same quantitative for both extraction processes (at 2 and 6 h): 50 g of inflorescences for *C. sativa*, 20 g of leaves and stems for *T. vulgaris* and 106 g of seeds for *C. sativum*. 250 mL of ethanol were used as extraction solvent. The 2 hours process (N2h) was carried out by processing plant matrix for 30 cycles (with a maximum pressure of 8 bar); each cycle was composed by 12 hits in the dynamic phase (2 min duration) and a duration of the static phase of 2 min. The 6 h extracts (N6h) were obtained with the same conditions, but with a major number of cycles (i.e., 90).

#### 2.3.2. Soxhlet Extraction

Soxhlet extracts were produced starting from the same quantitative for both extraction processes (at 2 and 6 h, S2h and S6h, respectively): 50 g of inflorescences for *C. sativa*, 20 g of leaves and stems for *T. vulgaris* and 106 g of seeds for *C. sativum* were used. The extractions were performed with 250 mL of ethanol at 100 °C.

#### 2.3.3. Maceration

Macerations were performed using 9 g of inflorescences for *C. sativa*, 4 g of leaves and stems for *T. vulgaris*, and 21 g of seeds for *C. sativum*. The macerates (M) were obtained with 50 mL of ethanol as solvent for 30 days at room temperature without light exposure.

#### 2.3.4. UAE Extraction

The UAE extractions were performed using 19 g of inflorescences for *C. sativa*, 8 g of leaves and little stems for *T. vulgaris* and 42 g of seeds for *C. sativum*. Plant matrices were extracted with 100 mL of ethanol in 250 mL flasks, sealed and immersed in an ultrasonic water bath (Argo Lab DU-45, Milan, Italy) for 15 min (40 kHz, 180 W).

### 2.4. SPME/GC–MS Characterization of Extracts Volatile Fraction

Chemical characterizations of extracts volatile fraction were performed by solid-phase microextraction/gas chromatography coupled to mass spectrometry (SPME/GC-MS). SPMEs were obtained by a Supelco-57299-U SPME DVB/CAR/PDMS (Divinylbenzene/Carboxen/Polydimethylsiloxane) fiber (Sigma Aldrich-Saint Louis, MO, USA). All extracts were processed as follows: 0.50 g of dry extract were put into a 20 mL capacity glass vial and sealed with a rubber septum and an aluminum. The vial was placed on a heated plate (50 °C) and the SPME needle was inserted into the vial. The grey fiber was exposed to the headspace for 20 min. After exposure, the fiber was retracted into a needle and loaded into the injection port of the gas chromatographer for fiber desorption at 250 °C for 15 min.

A Clarus 580 GC apparatus (PerkinElmer-Waltham, MA, USA) coupled to a Clarus SQ 8 S GC/MS (PerkinElmer-Waltham, MA, USA) was used for GC-MS analysis. Separations were achieved on a fused silica Zebron-ZB-SemiVolatile column (30 m × 250 μm × 0.25 μm—Phenomenex, Torrance, CA, USA). Analyses were carried following a different temperature gradient depending on samples.

The temperature gradient for hemp extracts was as follows: starting temperature 50 °C (hold 1 min), up to 145 °C at 7 °C/min (hold 5 min), up to 175 °C at 4 °C/min and up to 250 °C at 7 °C/min (hold 5 min). The carrier gas was Helium (flow rate 1 mL/min). The split of the injector was set to 1:50, while the injector and the transfer line temperature were set at 250 °C.

The temperature gradient for thyme and coriander extracts was as follows: starting temperature 45 °C (hold 10 min), up to 180 °C at 2.5 °C/min (hold 5 min). The carrier gas was Helium (flow 1 mL/min), while the injector and the transfer line temperature were set at 250 °C.

The semi-quantitative characterization was carried out through Turbomass 6.1.0.1963 software (PerkinElmer-Waltham, MA, USA). The unknown compounds were identified by matching the obtained spectra with the NIST Mass Spectral Library 2.0 (NIST-Gaithersburg, MD, USA) and confirmed by comparison of the retention index (RI) with those retrieved from http://webbook.nist.gov/chemistry/. A mix of *n*-alkanes, ranging from octane (C8) to triacontane (C30) was obtained from Supelco (Bellefonte, CA, USA) and injected using the analytical conditions above reported to determine the retention index (RI) as proposed by Lee et al. [20].

Semi-quantitative analysis was made by peak area normalization without response factors. Relative abundances (%) were the mean of two replicates.

### 2.5. HPLC-UV Characterization of the Phenolic Fraction

Phenolic compounds were determined by HPLC (Perkin-Elmer series 200, Monza, Italy) equipped with an autosampler and a UV-Vis detector (Perkin Elmer LC 240, Monza, Italy) set at 280 nm. For separation, a Phenomenex Kinetex C18 column was used (dimensions: 250 × 4.6 mm, particle size: 5 µm, pore size: 110 Å; Phenomenex, Bologna, Italy). The mobile phases used were: (A) 1% acetic acid in water and (B) acetonitrile. For analyte separation, the mobile phase gradient was programmed as follows: from 10% to 100% solvent B for 30 min and subsequent return to initial composition in 4 min, achieving mobile phase stabilization for 10 min.

40 mg of dry extracts sample was dissolved in 1 mL of water/methanol (50:50), vortexed for 3 minutes, centrifugated for 15 min, filtered with 0.2 µm PTFE filter and analyzed.

Quantification of polyphenols was carried out by the external standard method. Linear regression curves based on peak area were calculated for each phenolics compound after injection of mix phenolic standard solutions covering the sample range of concentrations (6-12-25-50-100 ppm).

For quantitative analysis, a calibration curve for each available phenolic standard were constructed based on the UV signal: gallic acid (y = 36,255x − 26,062; R^2^ = 0.9983), p-OH-benzoic (y = 31,711x − 16,966); R^2^ = 0.9992), vanillic acid (y = 33,123x − 52,417; R^2^ = 0.9974), rosmarinic acid (y = 34,344x − 40,066; R^2^ = 0.9992), ferulic acid (y = 61,245x + 74,735; R^2^ = 0.9912), caffeic acid (y = 44,841x − 416,813; 0.9952), syringic acid (y = 39,490x − 106,101; R^2^ = 0.9987), luteolin (y = 7593,1x − 19,075; R^2^ = 0.9991), apigenin (y = 83,755x − 7443,3; R^2^ = 0.9979), and chlorogenic acid (y = 29,136x − 63,864; R^2^ = 0.9971).

### 2.6. Total Phenolic Content (TPC) and Antioxidant Capacity (AOC)

Total Phenolic Content (TPC) estimation was carried out by means of Folin-Ciocâlteu’s reagent, following the Singelton and Rossi method [21]. The reference standard was gallic acid (GA). Results are expressed as mg GA equivalents (GAE)/g dry extract, mean value of two replicates.

The antioxidant activity (AOC) was investigated employing:

- DPPH˙ assay, following the method proposed Brand-Williams et al. [22];
- ABTS˙^+^ assay, with the Gullon et al. method [23],
- FRAP assay, assessed by means of potassium ferricyanide-ferric chloride method described by Oyaizu [24].

For FRAP, DPPH˙, and ABTS˙^+^ assays, Trolox was used as a reference standard. Results are expressed as mg Trolox equivalents (TE)/g dry extract, mean value of two replicates.

### 2.7. Statistical Analysis

Results were expressed as means ± standard deviations. Yields, chemical, and biological characterization data were subjected to ANOVA (analysis of variance), followed by Tukey’s HSD post-hoc test at a significance level of 5% (*p* < 0.05). Terpenes classes composition obtained by SPME/GC-MS were processed through principal component analysis (PCA) to observe the possible correlations within the extracts of the different matrices. Before applying the PCA algorithm, the data were linearized and automatically scaled (zero mean and unit variance) to eliminate the differences in the concentration range. The data set consisted of 18 × 4, in which rows represented the 18 extracts and columns the 4 terpenes classes. Data on terpenes classes were also treated using a hierarchical clustering method. Dendrograms were constructed using Euclidean distance measure and Ward’s method of dissimilarity between clusters. Both statistical tests were performed with Microsoft Xlstat 2016 statistical software (Addinsoft, Paris, France).

## 3. Results and Discussion

### 3.1. Yields

The extracts yields obtained for the three plant matrices are reported in Table 1. The highest yield for thyme was obtained for 2 h Soxhlet extraction (S2h), while the lowest for maceration (M) and ultrasound assisted extraction (UAE) (*p* < 0.05). The best yield for hemp was achieved, indeed, for 6 h Soxhlet extraction (S6h), while the lowest for M and UAE extracts (*p* < 0.05). For this matrix, no significant differences were recorded among RSLDE extraction times (N2h and N6h) and S2h (*p* > 0.05). A totally different behavior was observed for coriander seeds extracts: the best yield was found for UAE and, then M; the lowest was N2h.

According to these data, Soxhlet seems to be the most suitable technique for the extraction of plants aerial parts in terms of yield. However, it should be pointed out that this extraction technique carried out at high temperature allows the co-extraction of the fibers [25,26,27,28,29]. These contribute to the dry extract weight. On the other hand, ultrasound assisted method seems to be the best extraction technique to process plant seeds.

Lower yields were obtained for coriander seeds with respect to hemp and thyme. This is common to other plant species. In fact, the best yields of extraction are usually recovered from stems and leaves [30,31]. In any case, our findings are in line with literature data, falling within the intervals normally reported in several works for the same species for some of these techniques [28,32].

### 3.2. Chemical Composition of Extracts Volatile Fraction

The SPME/GC–MS characterization data of the volatile fraction of the extracts of the three plants are shown in Table 2. 

In thyme extracts 22 compounds were found, 21 monoterpenes, and one sesquiterpene. Thymol has generally been reported to be the main component of *T. vulgaris*. However, this cultivar contains carvacrol, the isomer of thymol, that has the same biological activity. Linalool was the most abundant volatile compound in all the extracts, but using Soxhlet for two hours, a much lower quantity was found. Both carvacrol and linalool are natural effective antimicrobials used to control the growth of spoilage microorganisms in food as demonstrated in some studies in literature [33,34]. They have been reported to have also therapeutic properties (e.g., vs. Alzheimer’s disease) [35].

A total of 25 compounds were identified in hemp extracts, 13 monoterpenes, and 12 sesquiterpenes. The predominant compounds were: β-myrcene and caryophyllene within the monoterpenes and sesquiterpenes, respectively. β-myrcene is known to possess anti-inflammatory, analgesic, and anxiolytic properties [36,37]. Caryophyllene has been reported as anti-inflammatory compound in some cannabis preparations because of the interaction with the cannabinoid receptors and a gastric cytoprotective activity has been also found [38,39,40]. Interestingly, caryophyllene oxide seems to be a multi-target molecule, known for its anticancer and analgesic properties [37].

Furthermore, 18 terpenoids were identified in coriander seeds extracts, all belonging to the monoterpenes class. The most abundant compound was linalool, which has antibacterial activity [33,34] and anti-tumorigenic potential [41]. Canfora and *cis*-geraniol were also present in smaller amounts; however, they have been reported to contribute to biological and antioxidant activity [37,42]. In literature, there are few studies of the chemical composition of extracts from coriander fruits; the terpenes profile found is similar to coriander seeds essential oils previously reported by Pellegrini et al. [43] and found in literature [44,45,46].

For each matrix, the SPME/GC-MS identified compounds were associated with four main classes of terpenes (class assignment of each compound is in Table 2). To explore potential correlations among the whole data set the PCA algorithm was used.

**Figure 1:**
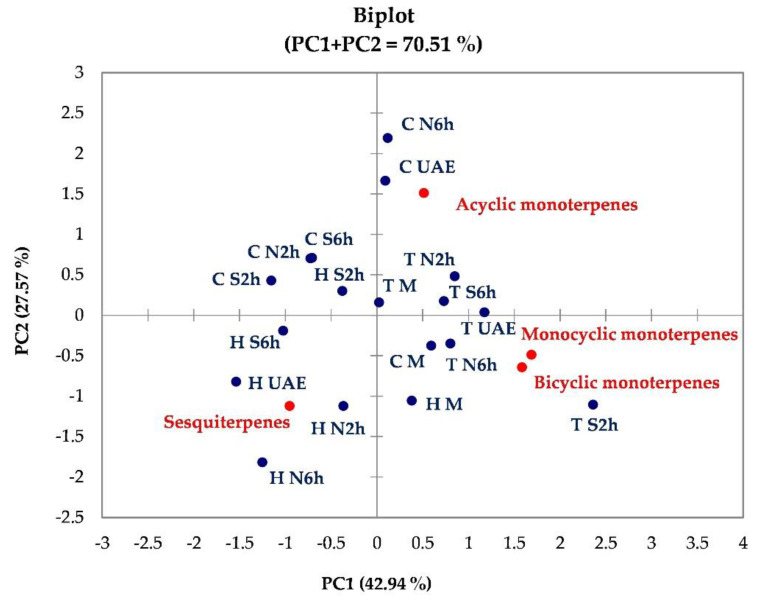
Biplot (scores and loadings) obtained from the PCA on data set of different extracts (rows) and terpenes classes analyzed (columns). In the Figure: T, thyme; H, hemp; C, coriander seeds; N2h, RSLDE 2 h; N6h, RSLDE 6 h; S2h, Soxhlet 2 h; S6h, Soxhlet 6 h; UAE, ultrasound assisted extraction; M, maceration.

Figure 1 reports the PCA biplot obtained for the different terpenes classes (loadings), determined in extracts of thyme, hemp, and coriander (scores). The total variance explained was 70.51%, with the first component accounting for 42.94% and the second for 27.57%.

From the biplot, it is evident that thyme extract obtained from Soxhlet at 2 h of extraction (T S2h) is separated from all extracts based on the major content of monocyclic and bicyclic monoterpenes. These two classes of terpenes are strongly correlated. Sesquiterpenes were the most abundant compounds in hemp 6 h RSLDE extract (H N6h), while acyclic monoterpenes represented mainly the volatile fractions of coriander ultrasounds-assisted (C UAE) and 6 h RSLDE (C N6h) extracts. Based on the studied variables, from PCA is also evident the presence of different clusters; in particular, on PC1 the extracts C M, T N6h, T S6h, and T N2h are well grouped (positive correlation with PC1). The same applies for C S6h, C S2h, C N2h, H S2h, and H S6h (negative correlation with PC1).

**Figure 2:**
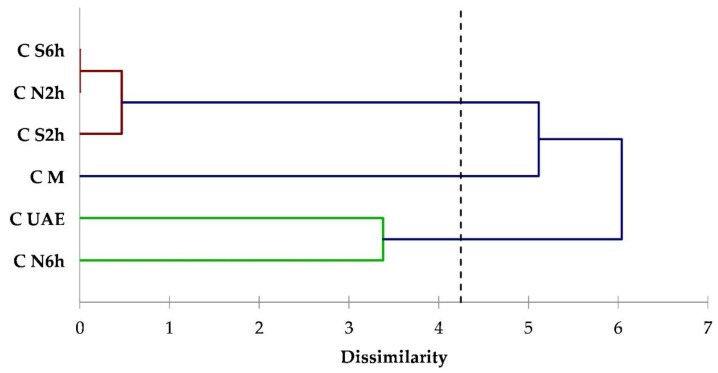
Dendrogram obtained from the cluster analysis based on terpenes classes data for coriander seeds extracts.

**Figure 3:**
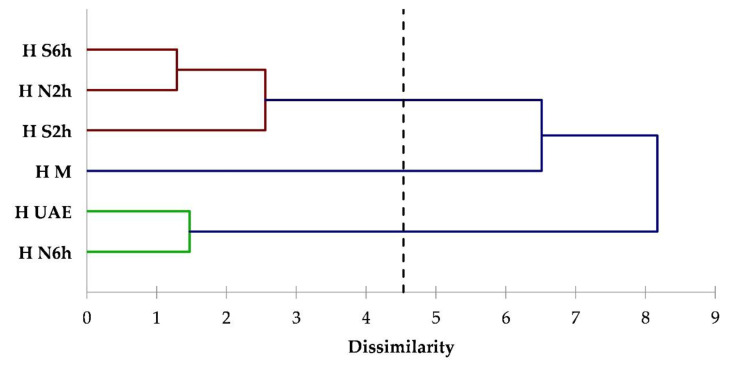
Dendrogram obtained from the cluster analysis based on terpenes classes data for hemp extracts.

**Figure 4:**
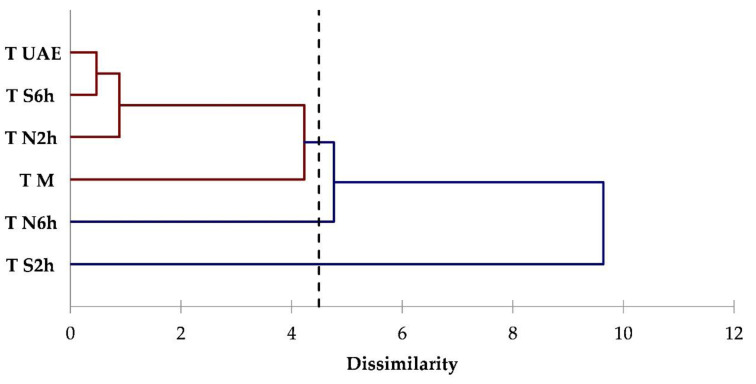
Dendrogram obtained from the cluster analysis based on terpenes classes data for thyme extracts.

To evaluate the influence of the extraction techniques on each matrix, the dataset was also processed through cluster analysis. Cluster analysis is a valid tool of multivariate analysis that has been already used and is useful to underline the differences among extraction techniques and conditions for the isolation of compounds from plant matrices [47,48]. Clusters were formed to contain four components (acyclic monoterpenes, monocyclic monoterpenes, bicyclic monoterpenes, sesquiterpenes). The dendrograms obtained from a cluster analysis of coriander, hemp, and thyme data are illustrated in Figure 2, Figure 3 and Figure 4, respectively.

From the coriander dendrogram (Figure 2) is evident that the diagram is divided into three classes: one class comprising only M extract, and the other 2 constituted by N2h and Soxhlet at 2 and 6 h (S2h and S6h) extracts, and N6h and UAE extracts, respectively. A similar classification was achieved for hemp (Figure 3). In both dendrograms, the N6h/UAE class has large distance from the M class, meaning that these extraction techniques allowed for the isolation of different classes of terpenes. Indeed, N2h and Soxhlet at both 2 and 6 h of distillation time, have similar extraction patterns.

For thyme (Figure 4), a different distribution is obtained; one class consists of M, N2h, S6h, and UAE, the second and third consisting of only N6h and S2h, respectively. In this case, a larger distance of S2h from the first group was observed, because of the ability of this technique to extract more monocyclic and bicyclic monoterpenes, as already evidenced by PCA (Figure 1).

The cluster analyses of terpenes allowed to underline that there were not clear patterns of extraction that can help in selecting a particular technique for the extraction of a certain class of terpenes. This is mainly related to the variables that occur before, during and after the extraction process and that influence the outcomes [8]. Anyhow, for all matrices the RSLDE at 2 h of extraction is always clustered with Soxhlet at 6 hours, indicating that, regardless of matrix nature, the extraction patterns are very similar for the two approaches.

### 3.3. Polyphenolic Composition

The HPLC-UV qualitative and quantitative analysis results of the extracts are presented in Table 3. 

Eight phenolic acids, (i.e., gallic acid, *p*-OH-benzoic acid, chlorogenic acid, vanillic acid, caffeic acid, syringic acid, ferulic acid, and rosmarinic acid), one phenolic monoterpene (carvacrol) and two flavonoids (i.e., luteolin and apigenin) were identified in thyme. Rosmarinic acid was the compound with the highest concentration in all extracts. Rosmarinic acid is known as one of the main constituents of thyme and it has been recognized for antioxidant, antiviral, anti-inflammatory, antibacterial, and immunostimulant activities [49,50]. UAE and M extracts were significantly poorer of polyphenols, except for the highest UAE luteolin content; however, they were the only extracts containing some phenolic acids (i.e., vanillic and caffeic acids in UAE, syringic acid in M). RSLDE exhibited improved extraction of *p*-OH-benzoic and chlorogenic acids. A non-univocal effect of the increase of the extraction time was observed, as some components have increased and others decreased. In line with our findings, the decrease in content of rosmarinic acid and luteolin at prolonged extraction time has been reported by other authors [51,52]. Besides the extraction operative conditions like solvent, temperature, and time, the stability of natural products in certain conditions is a variable that may influence the chemical composition of the extract. The bioactive compound, during the extraction procedure, are exposed to chemical reactions with solvent and/or other components in the solution that rearrange chemical structures. Chemical alterations occur also after the extraction process, due to manipulation (e.g., solvent removal) and/or conservation conditions (e.g., compounds breakdown by oxidation or light) [53,54].

Three phenolic acids (i.e., gallic acid, *p*-OH-benzoic acid, and rosmarinic acid) and two flavonoids (i.e., luteolin and apigenin) were mainly found in hemp. The most abundant component was luteolin, a flavonoid with antioxidant [55], anti-inflammatory and antiallergic [56] activity. Among the few literature studies on the identification of polyphenols in hemp extracts, the presence in hemp essential oil of gallic acid and *p*-OH benzoic acid has been already reported [57]. Considering the different extraction techniques, N2h was the extract richest in the three main compounds: rosmarinic acid, luteolin, and apigenin. N6h indicated that the increase in extraction time allowed the recovery of gallic acid, *p*-OH-benzoic acid, and ferulic acid, with a loss in the main components. Indeed, the increase in Soxhlet extraction time from 2 h to 6 h led to an increase in the concentration of gallic acid and of the two flavonoids luteolin and apigenin, accompanied by the decrease of *p*-OH-benzoic acid and rosmarinic acid. M was the poorest extract compared to other techniques.

Similarly to thyme, eight phenolic acids (i.e., gallic acid, *p*-OH-benzoic acid, chlorogenic acid, vanillic acid, caffeic acid, syringic acid, ferulic acid, and rosmarinic acid) and two flavonoids (i.e., luteolin and apigenin) were detected in coriander seeds. Barros and collaborators [58] reported a similar profile in the analysis of polyphenols of coriander seeds. However, gallic acid, luteolin, and apigenin were not detected. Msaada and collaborators [59] described a similar composition, with a higher number of flavonoids components (quercetin, rutin, luteolin, apigenin, and kaempferol). Among the different extraction techniques, RSLDE extracts were richer in gallic, *p*-OH-benzoic, and rosmarinic phenolic acids and in luteolin and apigenin, with a positive effect of longer extraction time on their concentration, except for *p*-OH-benzoic acid and luteolin. On the contrary, Soxhlet extracts showed higher concentrations of chlorogenic, vanillic, and caffeic acids, in addition to the presence of syringic acid, that was not detected in N2h and N6h.

### 3.4. Total Phenolic Content and Antioxidant Activity

Total phenolic content (TPC) and antioxidant activity (AOC-FRAP, DPPH˙, ABTS˙^+^) data are reported in Table 4. The different extraction techniques were compared using an analysis of variance (ANOVA).

The comparison with literature indicates that our data are in accordance with those reported by different authors [60,61,62,63,64].

The influence of the extraction time on TPC was more evident for thyme RSLDE extracts and coriander Soxhlet extracts, with improved extraction for longer times (6 h with respect to 2 h). In the comparison of the different techniques, the highest values were registered for RSLDE extracts for thyme and hemp, at 6 h and 2 h, respectively. Indeed, in the case of coriander seeds, Soxhlet extraction at 6 h was more efficient. For all plant matrices, UAE extracts had the lowest TPC values.

Concerning AOC (Table 4) of hemp and coriander seeds, a higher activity for the 2 h RSLDE extracts in all spectrophotometric assays was achieved, except for the DPPH˙. The highest antiradical activity was obtained for UAE extracts. Moreover, in the case of hemp, M demonstrated good activity, similarly to RSLDE extracts.

It is known that the differences in the antioxidant activity might be related to the different availability of extractable components, resulting from the varied chemical composition of plants [65]. The amount of the antioxidant components that can be extracted from a plant material is mainly affected by the strength of the extraction procedure and may vary from sample to sample. Usually, the TPC and AOC of extracts obtained by reflux extraction technique (e.g., Soxhlet) are lower than other methods [27], in contrast to the trends noted for extraction yields. This decrease can be attributed to the thermal decomposition of some antioxidants at the temperatures used in the process. Several studies reported that thermal processing conditions might result in the loss of natural antioxidants because heat may accelerate oxidation and other degradation reactions [27,66,67].

In this work, a different behavior was observed; for thyme, in fact, Soxhlet at 6 h was the best extract in terms of antioxidant activity. This indicates that for this aromatic plant, hot solvent systems under reflux state are more efficient for the recovery of antioxidant components. Dutra et al. [68] reported that among different extraction techniques (i.e., reflux, maceration, ultrasound, heating plate), extraction made under reflux using ethanol/water (70:30, *v*/*v*) offered the highest polyphenol levels in *Pterodon emarginatus* vogel seeds. This was attributed to the effective extraction under reflux conditions, leading to a higher release of some bound phenolics and with an increase of antioxidant activity [69].

To highlight the comparison of the different extraction techniques, for each plant matrix, the best performing extracts in TPC and antioxidant activity assays are collected in Table 5.

Table 5 clearly shows that all the extraction techniques are very efficient methods for the recovery of bioactive compounds from plant matrices, particularly to produce extracts retaining good antioxidant capacity. It is worth to notice, however, that more than half of the cases are carried out using unconventional techniques, (N2h/N6h and UAE), thus reducing solvent and energy consumption.

## 4. Conclusions

In this study, extracts have been obtained from three plant species *T. vulgaris*, *C. sativa,* and *C. sativum* cultivated in the Abruzzo region. Four different extraction methods were applied to recover the plants’ bioactive components, two conventional, namely the maceration and Soxhlet technique, and two unconventional, namely the ultrasound assisted extraction and the rapid solid–liquid dynamic extraction, performed by Naviglio Extractor^®^. Moreover, for the Soxhlet and RSLDE techniques, the effect of the extraction time was also investigated.

All the obtained extracts are rich in bioactive compounds and display good antioxidant properties. Although the results do not show univocal trends, slightly higher performances are observed for the extracts obtained by the unconventional RSLDE and UAE techniques.

Given the limited effect of the increase in extraction time, the RSLDE technique performed by Naviglio Extractor^®^ at 2 h of extraction time can be considered a good standardized method to obtain extracts with interesting in vitro antioxidant activity and potential candidates as natural preservatives in food.

## Figures and Tables

**Table 1:** Yields of extracts (% *w*/*w*). N2h, RSLDE 2 h; N6h, RSLDE 6 h; S2h, Soxhlet 2 h; S6h, Soxhlet 6 h; UAE, ultrasound assisted extraction; M, maceration.

	N2h	N6h	S2h	S6h	UAE	M
**Thyme**	2.30 ± 0.06 d	2.45 ± 0.09 c	9.25 ± 0.02 a	8.65 ± 0.05 b	1.62 ± 0.02 e	1.78 ± 0.04 e
**Hemp**	6.00 ± 0.03 b	5.81 ± 0.05 b	5.70 ± 0.01 b	10.00 ± 0.07 a	0.71 ± 0.09 c	0.95 ± 0.04 c
**Coriander seeds**	0.57 ± 0.09 f	0.73 ± 0.08 e	1.18 ± 0.06 d	1.63 ± 0.09 c	2.36 ± 0.07 a	2.17 ± 0.08 b

Results followed by the same case-letter are not different according to Tukey’s HSD post-hoc test (*p* > 0.05).

**Table 2:** Solid-phase microextraction/gas chromatography-mass spectrometry (SPME/GC-MS) characterization of the volatile fraction of thyme, hemp and coriander seeds extracts. Results are expressed as relative abundances % (means ± sd).

**ID-Thyme**	**Terpenes Class**	**RI**	**N2h**	**N6h**	**S2h**	**S6h**	**UAE**	**M**
α-pinene	bicyclic monoterpenes	921	2.11 ± 0.73	2.34 ± 0.31	2.53 ± 0.19	2.26 ± 0.10	2.47 ± 0.35	1.57 ± 0.16
sabinene	bicyclic monoterpenes	967	2.49 ± 0.61	2.82 ± 0.27	2.36 ± 0.09	2.90 ± 0.22	3.04 ± 0.22	2.21 ± 0.25
1-octen-3-ol	acyclic monoterpenes	980	0.63 ± 0.02	0.31 ± 0.01	0.30 ± 0.00	0.61 ± 0.04	0.82 ± 0.13	0.22 ± 0.00
β-myrcene	acyclic monoterpenes	999	0.52 ± 0.02	0.56 ± 0.08	0.82 ± 0.23	0.60 ± 0.01	0.56 ± 0.03	0.43 ± 0.00
ɣ-3-carene	bicyclic monoterpenes	1010	3.52 ± 0.38	4.20 ± 0.94	3.26 ± 0.96	4.16 ± 0.04	4.10 ± 0.19	3.15 ± 0.15
*o*-cymene	monocyclic monoterpenes	1019	0.18 ± 0.01	0.90 ± 0.08	1.24 ± 0.54	0.45 ± 0.08	1.31 ± 0.31	0.16 ± 0.01
*p*-cymene	monocyclic monoterpenes	1023	1.18 ± 0.04	1.33 ± 0.20	7.81 ± 0.26	1.38 ± 0.10	1.34 ± 0.03	1.01 ± 0.04
ɣ-terpinene	monocyclic monoterpenes	1054	5.45 ± 0.49	3.21 ± 0.54	5.48 ± 0.16	7.17 ± 0.80	6.81 ± 0.19	5.03 ± 0.22
*cis*-sabinene hydrate	bicyclic monoterpenes	1066	5.93 ± 0.76	4.88 ± 0.26	2.29 ± 0.53	5.06 ± 0.42	5.96 ± 0.05	4.14 ± 0.09
*cis*-linalool oxide	monocyclic monoterpenes	1079	1.14 ± 0.14	1.30 ± 0.27	5.68 ± 0.33	1.33 ± 0.06	1.20 ± 0.04	1.03 ± 0.01
linalool	acyclic monoterpenes	1096	48.19 ± 1.95	46.32 ± 1.23	16.19 ± 2.88	42.59 ± 1.37	47.66 ± 3.79	44.95 ± 1.81
*cis*-*p*-menth-2-en-1-ol	monocyclic monoterpenes	1123	2.03 ± 0.65	0.81 ± 0.02	14.36 ± 0.19	0.37 ± 0.05	0.68 ± 0.01	0.19 ± 0.01
*trans*-limonene oxide	monocyclic monoterpenes	1138	2.20 ± 0.54	0.89 ± 0.11	1.24 ± 1.68	0.15 ± 0.02	0.19 ± 0.07	0.40 ± 0.00
β-pinene oxide	bicyclic monoterpenes	1155	0.01 ± 0.00	1.06 ± 0.01	6.92 ± 0.86	0.36 ± 0.04	0.79 ± 0.02	0.30 ± 0.00
*trans*-linalool oxide	monocyclic monoterpenes	1174	5.09 ± 0.01	5.00 ± 0.76	6.36 ± 0.67	5.25 ± 0.25	6.75 ± 0.24	3.24 ± 0.27
α-terpineol	monocyclic monoterpenes	1190	3.07 ± 0.46	3.46 ± 0.47	1.44 ± 0.28	3.57 ± 0.12	4.28 ± 1.17	3.54 ± 0.11
*trans*-piperitol	monocyclic monoterpenes	1208	0.71 ± 0.14	0.44 ± 0.03	4.76 ± 0.65	0.19 ± 0.00	0.21 ± 0.03	0.19 ± 0.15
6,7-epoxigeranial	acyclic monoterpenes	1232	0.76 ± 0.03	0.65 ± 0.01	0.85 ± 0.15	0.39 ± 0.11	0.26 ± 0.10	0.32 ± 0.01
carvone	monocyclic monoterpenes	1243	0.76 ± 0.01	0.3 ± 0.01	0.05 ± 0.01	0.66 ± 0.06	1.01 ± 0.42	0.45 ± 0.02
linalyl acetate	acyclic monoterpenes	1247	2.49 ± 0.20	1.71 ± 0.64	2.74 ± 0.70	3.35 ± 0.27	2.82 ± 0.69	2.56 ± 0.14
carvacrol	monocyclic monoterpenes	1296	9.18 ± 2.01	15.84 ± 1.09	6.52 ± 2.26	13.99 ± 2.68	5.49 ± 0.75	21.30 ± 0.72
β-bisabolene	sesquiterpenes	1508	1.29 ± 0.31	0.67 ± 0.01	2.00 ± 0.49	1.50 ± 0.23	1.21 ± 0.50	1.70 ± 0.08
**D-Hemp**	**Terpenes Class**	**RI**	**N2h**	**N6h**	**S2h**	**S6h**	**UAE**	**M**
α-thuyene	bicyclic monoterpenes	898	0.07 ± 0.00	0.06 ± 0.02	0.02 ± 0.0	0.02 ± 0.0	-	0.13 ± 0.01
α-pinene	bicyclic monoterpenes	915	0.09 ± 0.00	0.10 ± 0.04	0.40 ± 0.1	0.16 ± 0.1	-	0.22 ± 0.04
β-pinene	bicyclic monoterpenes	959	0.16 ± 0.01	0.15 ± 0.00	0.34 ± 0.0	0.14 ± 0.0	0.28 ± 0.03	0.20 ± 0.03
β-myrcene	acyclic monoterpenes	969	2.47 ± 0.29	0.78 ± 0.11	4.27 ± 0.04	3.03 ± 0.8	-	1.98 ± 0.14
D-limonene	monocyclic monoterpenes	1011	0.65 ± 0.34	0.26 ± 0.02	0.55 ± 0.01	0.36 ± 0.0	0.10 ± 0.03	0.61 ± 0.05
eucaliptol	bicyclic monoterpenes	1016	0.86 ± 0.41	0.46 ± 0.07	1.01 ± 0.0	0.74 ± 0.1	0.11 ± 0.02	2.14 ± 0.26
β-ocymene	acyclic monoterpenes	1027	0.95 ± 0.65	0.15 ± 0.04	2.27 ± 0.1	1.11 ± 0.3	2.25 ± 0.08	0.52 ± 0.01
ɣ-terpinene	monocyclic monoterpenes	1039	0.41 ± 0.15	0.89 ± 0.11	0.46 ± 0.00	0.25 ± 0.00	0.32 ± 0.05	1.66 ± 0.07
terpinolene	monocyclic monoterpenes	1067	3.75 ± 0.17	0.37 ± 0.04	3.79 ± 0.03	5.05 ± 0.9	0.03 ± 0.00	2.15 ± 0.72
linalool	acyclic monoterpenes	1079	4.93 ± 0.30	9.42 ± 1.67	2.69 ± 0.04	0.63 ± 0.0	0.26 ± 0.04	25.98 ± 1.73
L-*trans*-pinocarveol	bicyclic monoterpenes	1109	0.87 ± 0.36	0.75 ± 0.01	0.42 ± 0.1	0.60 ± 0.1	0.81 ± 0.15	0.60 ± 0.06
*cis*-*p*-mentha-2,8-dien-1-ol	monocyclic monoterpenes	1279	1.58 ± 0.39	1.23 ± 0.10	0.92 ± 0.2	1.35 ± 0.0	0.23 ± 0.04	1.20 ± 0.01
geranyl acetate	acyclic monoterpenes	1372	1.00 ± 0.16	0.72 ± 0.01	1.46 ± 0.59	1.53 ± 0.36	0.96 ± 0.63	1.50 ± 0.18
caryophyllene	sesquiterpenes	1390	51.85 ± 2.57	52.78 ± 2.61	52.44 ± 0.1	54.78 ± 0.1	40.00 ± 0.36	39.61 ± 3.31
α-bergamotene	sesquiterpenes	1400	5.86 ± 1.26	6.14 ± 0.51	4.83 ± 0.0	6.53 ± 0.2	4.79 ± 0.22	2.56 ± 0.52
*cis*-β-farnesene	sesquiterpenes	1416	3.82 ± 0.50	3.54 ± 0.42	2.70 ± 0.1	4.20 ± 0.3	4.03 ± 0.35	2.18 ± 0.18
humulene	sesquiterpenes	1423	13.60 ± 1.81	13.49 ± 0.14	12.11 ± 0.2	14.13 ± 0.4	12.25 ± 0.99	8.67 ± 0.05
aromadendrene	sesquiterpenes	1428	1.85 ± 0.18	2.86 ± 0.12	2.35 ± 0.0	2.09 ± 0.1	3.16 ± 0.19	2.55 ± 0.01
β-selinene	sesquiterpenes	1459	1.96 ± 0.79	2.81 ± 0.17	2.39 ± 0.1	0.96 ± 0.6	0.53 ± 0.10	3.37 ± 0.39
α-selinene	sesquiterpenes	1466	1.14 ± 0.86	1.91 ± 0.15	1.63 ± 0.0	1.12 ± 0.1	3.26 ± 0.59	2.30 ± 0.25
ɣ-cadinene	sesquiterpenes	1408	0.32 ± 0.11	0.20 ± 0.00	0.40 ± 0.0	0.41 ± 0.0	0.37 ± 0.07	0.27 ± 0.03
guaia-3–9-diene	sesquiterpenes	1413	1.08 ± 0.11	0.80 ± 0.09	1.60 ± 0.0	0.86 ± 0.1	0.00 ± 0.00	0.70 ± 0.09
selina-3,7(11)-diene	sesquiterpenes	1418	0.91 ± 0.08	0.48 ± 0.01	1.65 ± 0.1	0.80 ± 0.1	1.19 ± 0.21	0.47 ± 0.08
caryophyllene oxide	sesquiterpenes	1458	0.29 ± 0.04	0.27 ± 0.01	0.23 ± 0.0	0.29 ± 0.1	0.17 ± 0.03	0.20 ± 0.02
*cis*-α-bisabolol	sesquiterpenes	1589	0.05 ± 0.01	0.09 ± 0.07	0.04 ± 0.0	0.06 ± 0.00	0.32 ± 0.06	0.05 ± 0.00
**ID-Coriander Seeds**	**Terpenes Class**	**RI**	**N2h**	**N6h**	**S2h**	**S6h**	**UAE**	**M**
α-pinene	bicyclic monoterpenes	915	0.15 ± 0.04	0.33 ± 0.02	0.49 ± 0.07	0.15 ± 0.04	0.50 ± 0.01	0.34 ± 0.03
2-carene	bicyclic monoterpenes	920	0.04 ± 0.01	0.07 ± 0.02	0.21 ± 0.01	0.04 ± 0.01	0.39 ± 0.01	0.04 ± 0.01
*p*-mentha-1,3,8-triene	monocyclic monoterpenes	980	0.04 ± 0.01	0.05 ± 0.01	1.40 ± 0.03	0.04 ± 0.01	-	0.49 ± 0.01
β-terpinyl-acetate	monocyclic monoterpenes	1343	0.04 ± 0.01	0.10 ± 0.00	0.04 ± 0.01	0.04 ± 0.01	-	0.13 ± 0.02
eucaliptol	bicyclic monoterpenes	935	-	-	0.24 ± 0.03	-	0.33 ± 0.00	0.03 ± 0.00
β-myrcene	acyclic monoterpenes	987	0.14 ± 0.03	0.21 ± 0.00	2.21 ± 0.01	0.15 ± 0.03	0.98 ± 0.01	0.23 ± 0.02
β-ocymene	acyclic monoterpenes	1027	0.05 ± 0.01	0.09 ± 0.01	0.02 ± 0.01	0.05 ± 0.01	-	0.04 ± 0.01
ɣ-terpinene	monocyclic monoterpenes	1053	0.15 ± 0.02	0.08 ± 0.01	2.24 ± 0.03	0.16 ± 0.02	1.41 ± 0.00	1.12 ± 0.01
*trans*-linalool oxide	monocyclic monoterpenes	1083	0.74 ± 0.03	0.59 ± 0.01	7.50 ± 0.04	0.75 ± 0.03	0.55 ± 0.04	2.09 ± 0.01
terpinolene	monocyclic monoterpenes	1085	0.04 ± 0.01	0.06 ± 0.01	0.03 ± 0.01	0.04 ± 0.01	-	0.03 ± 0.01
borneol	bicyclic monoterpenes	1162	0.45 ± 0.03	0.39 ± 0.02	0.38 ± 0.06	0.45 ± 0.03	-	1.65 ± 0.01
linalool	acyclic monoterpenes	1079	84.16 ± 0.56	72.55 ± 0.03	54.73 ± 0.05	83.99 ± 0.56	82.95 ± 0.05	78.20 ± 0.02
canfora	bicyclic monoterpenes	1144	6.41 ± 0.05	3.26 ± 0.02	3.11 ± 0.45	6.47 ± 0.05	1.87 ± 0.03	7.90 ± 0.02
terpinen-4-ol	monocyclic monoterpenes	1173	0.66 ± 0.02	0.78 ± 0.00	6.05 ± 0.04	0.67 ± 0.02	0.47 ± 0.02	0.76 ± 0.01
α-terpineol	monocyclic monoterpenes	1190	1.05 ± 0.02	2.14 ± 0.03	13.30 ± 0.04	1.06 ± 0.02	5.65 ± 0.04	1.19 ± 0.01
*cis*-geraniol	acyclic monoterpenes	1248	3.96 ± 0.14	13.08 ± 0.02	0.25 ± 0.01	4.00 ± 0.14	0.67 ± 0.04	4.25 ± 0.04
lavandulyl acetate	acyclic monoterpenes	1270	1.42 ± 0.03	5.50 ± 0.01	0.77 ± 0.05	1.44 ± 0.03	1.44 ± 0.03	1.28 ± 0.02
geranyl acetate	acyclic monoterpenes	1372	0.48 ± 0.03	0.70 ± 0.02	2.77 ± 0.01	0.49 ± 0.03	0.58 ± 0.03	0.22 ± 0.01

In the table: ID, component name; RI, retention index; N2h, RSLDE 2 h; N6h, RSLDE 6 h; S2h, Soxhlet 2 h; S6h, Soxhlet 6 h; UAE, ultrasound assisted extraction; M, maceration.

**Table 3:** Contents of phenolic compounds (µg/g dry extract).

**Thyme**						
	**N2h**	**N6h**	**S2h**	**S6h**	**UAE**	**M**
Gallic acid	42.74 ± 0.50 d	58.73 ± 0.90 b	678.67 ± 0.40 a	48.78 ± 0.32 c	-	40.23 ± 0.89 e
*p*-OH-benzoic acid	190.47 ± 1.02 b	197.15 ± 0.89 a	-	148.68 ± 0.98 c	55.04 ± 0.95 d	17.97 ± 1.02 e
Chlorogenic acid	66.54 ± 0.96 c	120.44 ± 0.75 a	30.07 ± 0.89 d	97.21 ± 0.98 b	-	-
Vanillic acid	-	-	-	-	376.27 ± 0.56 a	-
Caffeic acid	-	-	-	-	145.92 ± 0.85 a	-
Syringic acid	-	-	-	-	-	159.69 ± 1.02 a
Ferulic acid	-	139.24 ± 0.96b	-	516.02 ± 0.84 a	-	-
Rosmarinic acid	34201.41 ± 1.05 b	33955.20 ± 1.02 c	51686.96 ± 0.95 a	31549.93 ± 1.25 d	15049.48 ± 1.09 e	261.55 ± 0.82 f
Luteolin	2671.96 ± 1.10 c	1554.86 ± 1.05 f	1931.34 ± 0.95 d	1704.21 ± 1.32 e	4143.43 ± 0.65 b	2099.47 ± 0.84 a
Apigenin	6608.97 ± 1.15 b	5309.40 ± 1.03 c	2909.13 ± 1.01 e	7618.77 ± 0.98 a	4416.70 ± 0.87 d	-
Carvacrol	3499.84 ± 1.15 b	885.03 ± 1.02 d	2873.81 ± 0.99 c	5595.41 ± 1.05 a	-	220.49 ± 0.98 e
**Hemp**						
Gallic acid	-	52.29 ± 0.98 c	118.07 ± 0.32 b	408.92 ± 0.63 a	35.10 ± 0.98 d	36.02 ± 0.65 d
*p*-OH-benzoic acid	-	47.70 ± 0.75 c	95.18 ± 0.95 a	36.42 ± 0.35 d	-	52.41 ± 0.96 b
Chlorogenic acid	-	-	-	-	-	-
Vanillic acid	-	-	-	-	-	-
Caffeic acid	-	-	36.98 ± 0.48 b	-	81.81 ± 0.91 a	-
Syringic acid	-	-	-	-	57.28 ± 0.64 a	-
Ferulic acid	-	247.77 ± 0.64 a	-	-	-	96.70 ± 0.93 b
Rosmarinic acid	259.56 ± 0.97 c	27.09 ± 0.85 f	206.30 ± 0.94 d	152.06 ± 0.65 e	514.33 ± 1.01 a	328.21 ± 1.10 b
Luteolin	1572.05 ± 1.04 a	304.37 ± 1.10 e	502.83 ± 0.95 d	753.01 ± 0.84 c	1384.09 ± 1.09 b	127.67 ± 1.03 f
Apigenin	72.99 ± 1.02 a	51.43 ± 0.48 c	35.77 ± 0.95 d	54.22 ± 1.06 b	-	-
Carvacrol	-	-	-	-	-	-
**Coriander Seeds**						
Gallic acid	42.37 ± 0.98 b	49.05 ± 0.65 a	22.45 ± 0.35 d	22.73 ± 0.36 d	-	31.02 ± 0.39 c
*p*-OH-benzoic acid	274.83 ± 0.95 a	31.80 ± 0.98 d	-	89.22 ± 0.84 b	-	46.61 ± 0.91 c
Chlorogenic acid	74.80 ± 0.67 e	480.49 ± 0.92 b	149.47 ± 0.41 c	490.56 ± 0.35 a	146.90 ± 0.83 d	27.82 ± 0.94 f
Vanillic acid	120.27 ± 0.87 e	208.65 ± 0.80 c	440.90 ± 1.25 a	273.23 ± 1.65 b	205.46 ± 0.96 d	-
Caffeic acid	-	208.66 ± 0.85 b	440.17 ± 0.75 a	54.21 ± 0.65 c	27.88 ± 0.35 d	-
Syringic acid	-	-	24.09 ± 0.35 b	65.20 ± 0.92 a	-	23.56 ± 0.24 b
Ferulic acid	188.92 ± 0.95 b	78.81 ± 0.85 c	20.64 ± 0.77 e	239.21 ± 0.8 7a	22.75 ± 0.64 d	23.37 ± 0.81 d
Rosmarinic acid	81.05 ± 1.05a	82.13 ± 0.97a	34.78 ± 0.91b	31.00 ± 0.85c	21.97 ± 0.98c	-
Luteolin	324.50 ± 0.94a	295.82 ± 1.12b	172.40 ± 1.18c	86.90 ± 0.98e	152.01 ± 1.32d	-
Apigenin	182.67 ± 1.20a	182.04 ± 1.15a	30.09 ± 1.06d	35.81 ± 1.14b	101.07 ± 1.23e	87.31 ± 0.98c
Carvacrol	-	-	-	-	-	-

Results followed by the same case-letter are not different according to Tukey’s HSD post-hoc test (*p* > 0.05).

**Table 4:** Total phenolic content (TPC) and antioxidant activity. Ferric Reducing Antioxidant Power (FRAP), and Trolox Equivalent Antioxidant Capacity with 2,2-diphenyl-1-picrylhydrazyl (DPPH˙), and 2,2′-azinobis-(3-ethylbenzothiazoline-6-sulfonic acid (ABTS˙^+^) of extracts of different techniques.

	TPC (mg GAE/g Dry Extract)	FRAP (mg TE/g Dry Extract)	DPPH˙ (mg TE/g Dry Extract)	ABTS˙^+^ (mg TE/g Dry Extract)
**Thyme**				
N2h	141.86 ± 7.85 b	63.86 ± 0.29 b	108.17 ± 0.08 a	23.62 ± 8.20 d
N6h	179.67 ± 12.71 a	66.95 ± 5.74 b	108.52 ± 0.16 a	52.12 ± 0.11 bc
S2h	157.86 ± 1.71 b	68.48 ± 1.23 ab	108.57 ± 0.08 a	49.07 ± 0.11 c
S6h	154.26 ± 11.23 b	73.53 ± 1.29 a	107.19 ± 1.22 a	134.46 ± 0.22 a
UAE	107.91 ± 9.01 c	40.60 ± 1.04 c	98.50 ± 1.71 b	53.72 ± 0.34 bc
M	142.08 ± 1.69 b	40.25 ± 2.93 c	92.60 ± 4.33 c	57.56 ± 0.34 b
**Hemp**				
N2h	140.25 ± 2.56 a	81.073 ± 2.31ab	34.02 ± 1.86 b	557.16 ± 6.57 a
N6h	139.52 ± 2.49 a	80.21 ± 1.76 abc	33.76 ± 0.89 b	485.10 ± 4.16 b
S2h	120.55 ± 5.42 b	74.66 ± 0.81 d	29.92 ± 0.32 d	394.39 ± 3.41 c
S6h	124.25 ± 3.61 b	78.69 ± 1.88 bc	31.54 ± 0.08 cd	433.22 ± 19.81 bc
UAE	110.30 ± 3.71 c	77.22 ± 0.92 cd	45.04 ± 1.23 a	381.26 ± 9.05 c d
M	125.12 ± 3.54 b	83.14 ± 1.63 a	32.43 ± 0.32 bc	502.16 ± 5.62 ab
**Coriander Seeds**				
N2h	19.87 ± 1.52 b	18.43 ± 0.15 a	147.60 ± 3.97 b	48.05 ± 1.60 a
N6h	17.67 ± 0.47 c	12.69 ± 0.39 d	163.55 ± 2.94 b	46.48 ± 1.78 a
S2h	15.54 ± 0.57 d	15.22 ± 0.39 bc	128.84 ± 3.94 b	21.90 ± 2.00 b
S6h	24.36 ± 1.01 a	16.91 ± 2.34 ab	150.61± 4.92 b	23.85 ± 1.60 b
UAE	3.01 ± 0.61 e	13.45 ± 1.26 cd	219.95 ± 2.44 a	1.12 ± 0.14 d
M	18.95 ± 0.11 bc	14.40 ± 0.09 cd	177.23 ± 1.46 ab	5.64 ± 1.04 c

Results followed by the same case-letter are not different according to Tukey’s HSD post-hoc test (*p* > 0.05).

**Table 5:** Extracts with the highest value of total phenolic content and antioxidant activity

	TPC	FRAP	DPPH˙	ABTS˙^+^
Thyme	N6h	S6h	N2h/N6h/S2h/S6h	S6h
Hemp	N2h/N6h	M	UAE	N2h
Coriander seeds	S6h	N2h	UAE	N2h/N6h
